# The emerging dialogue between synthetic genomics and clinical research

**DOI:** 10.1002/ctm2.70738

**Published:** 2026-07-07

**Authors:** Yue Liu, Ying‐Jin Yuan

**Affiliations:** ^1^ State Key Laboratory of Synthetic Biology School of Synthetic Biology and Biomanufacturing Tianjin University Tianjin China; ^2^ Frontiers Science Center for Synthetic Biology (Ministry of Education) Tianjin University Tianjin China

## 1 THE 'READ–EDIT–WRITE' FRAMEWORK: ADVANCING MEDICINE THROUGH GENOMIC TECHNOLOGIES

The ‘read–edit–write’ paradigm delineates the central trajectory of advances in life science and biotechnology. The ‘read’—driven by breakthroughs in genome sequencing—has profoundly influenced modern medicine and clinical research. High‐throughput sequencing has dramatically deepened our understanding of genome architecture and function, enabling a shift in disease investigation from symptom‐based diagnosis to precise molecular and mechanistic insights.^1^ It has enabled researchers to systematically uncover driver mutations in human cancers, pinpoint pathogenic variants for rare genetic disorders, and map genetic susceptibility loci, thereby establishing a robust foundation for personalized medicine, target‐based drug discovery and clinical decision‐making.

The subsequent emergence of genome editing technologies has enabled precise manipulation of the genome, from correcting pathogenic mutations to modulating gene regulatory networks, thereby opening new possibilities for therapeutic intervention‐the ‘edit’.^2^ Genome‐editing tools exemplified by the CRISPR–Cas systems have demonstrated remarkable clinical potential, from curing monogenic diseases such as sickle cell disease and *β*‐thalassemia to engineering T cells (CAR‐T) that have generated groundbreaking outcomes in hematologic malignancies and autoimmune diseases.^3^ Collectively, these advances mark a pivotal transition in clinical medicine: a shift from reading the genome to actively modifying it.

Building on these advances, synthetic biology introduces a bottom‐up perspective that provides an entirely new conceptual and technological framework for biomedical and clinical research. By enabling modular, programmable, and large‐scale redesign of genetic elements and cellular functions, synthetic biology expands toolkits for functional genomics, disease modeling, cell and gene therapies, and the development of next‐generation biomedical interventions. Extending the trajectory from gene editing to large‐scale rational genome design and engineering, these advances hold the promise of providing clinical research with powerful new approaches for mechanistic investigation and therapeutic innovation, driving disease modeling, diagnosis and treatment development toward a more precise and system‐level understanding of human biology and disease.

## 2 HUMAN GENOME SYNTHESIS TO RESEARCH AND BEYOND

Synthetic genomics, a cornerstone of synthetic biology, aims to predictably design and construct DNA that enables cells to acquire novel biological functions. The advancement of genome synthesis has enabled the de novo construction of viral and bacterial genomes as well as complete eukaryotic chromosomes, laying the technological foundation for extending genome writing to mammalian systems.^4^ Since the launch of the GP‐write initiative in 2015, the field has entered a new phase focused on advancing the synthesis of higher‐eukaryotic genomes, including the human genome. Within this framework, scientists have emphasized several critical technological challenges, including de novo genome assembly of large and repetitive genomic regions, cross‐species delivery of megabase‐scale DNA molecules, and functional reconstitution of synthetic genomes in recipient cells.^5^

Efforts to synthesize the human genome are accelerating worldwide, driven by the expectation that this emerging capability will help tackle questions relevant to human health and diseases that remain challenging beyond current methods and may ultimately redefine the paradigm of disease‐centric research and treatment. Achieving this goal requires developing tools and methods for constructing human chromosomes, thereby deepening our understanding of genome biology and broadening the future landscape of biotechnology and medicine.

**FIGURE 1:**
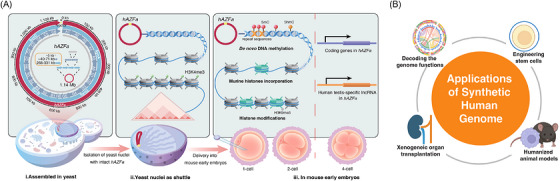
(A) Schematic of the SynNICE method (adapted from REF. [6]) and (B) Applications of the synthetic human genome (Figure created using biorender.com).

In our recent study, we developed SynNICE (Figure 1a), a method that integrates de novo assembly of megabase‐scale synthetic human DNA in yeast with cross‐species delivery into mammalian embryos.^6^ As a proof of concept, we chemically synthesized and assembled the 1.14‐Mb human *AZFa* region, which contains 69% repetitive sequences—substantially higher than the genomic average of 54%, and microdeletions of which cause the most severe male infertility. Using Nucleus Isolation for Chromosome Extraction (NICE), we isolated intact yeast nuclei that carry the synthetic chromosome, preserving chromatin architecture without cross‐linking.^7^ When the synthetic, naïve *hAZFa* construct—lacking any mammalian epigenetic marks—was microinjected into mouse early embryos, we observed de novo DNA methylation enriched at repetitive sequences which contributed to subsequent transcriptional regulation. The SynNICE method decouples DNA size constraints from delivery efficiency, enabling the construction of synthetic human chromosomes and facilitating deeper investigation into the mechanisms underlying the establishment of de novo epigenetic regulation.^8^ These advances provide a foundational framework for developing more faithful models of epigenetic dysregulation in human disease, early embryonic development and regenerative biology.

## 3 FUTURE OUTLOOK

Looking ahead, continued breakthroughs in foundational biotechnologies, together with anticipated reductions in the cost of large‐scale DNA synthesis, are poised to bring synthetic genomics into mainstream biomedical and clinical research. As the capability to design and build human genomic sequences becomes increasingly accessible, several transformative applications are expected to emerge (Figure 1b).

First, researchers will be able to reconstruct disease‐relevant human gene clusters with high precision—such as *HoxA*, *AZFa* and *DMD—*and introduce them into mouse models or organoids to recreate pathogenic and epigenetic abnormalities. By systematically designing, synthesizing and perturbing these regulatory regions, synthetic genomics will enable functional genomics at a mechanistic scale and in unprecedented depth, deepening our understanding of disease pathways and improving the validation efficiency of potential therapeutic targets. Second, synthetic genomics will accelerate progress in xenogeneic organ transplantation.^9^ By enabling rational genome‐scale modification, it will become feasible to engineer donor organs with improved immunological compatibility and enhanced safety, addressing one of the most pressing challenges in transplantation medicine. Third, the ability to engineer stem cells at much larger genomic scales will support the creation of more complex and traceable therapeutic cell products. This will open the door to next‐generation diagnostic and treatment strategies that integrate programmable circuits, lineage tracing and controlled differentiation, offering new possibilities for regenerative medicine.

Together, these developments highlight the potential of synthetic genomics to reshape the future landscape of clinical research—from mechanism‐focussed investigations to translational applications—ultimately enabling more precise, systematic and programmable approaches to understanding and treating human disease.

## CONFLICT OF INTEREST STATEMENT

The authors declare no conflicts of interest.

## Data Availability

The data that support the findings of this study are available on request from the corresponding author. The data are not publicly available due to privacy or ethical restrictions.
